# To dine in or not to dine in: A comparison of food selection and preparation behaviours in those with and without food security

**DOI:** 10.1002/hpja.427

**Published:** 2020-10-20

**Authors:** Lucy M. Butcher, Therese A. O’Sullivan, Maria M. Ryan, Johnny Lo, Julie Nyanjom, Hugh C. Wilkins, Amanda Devine

**Affiliations:** ^1^ Edith Cowan University Joondalup WA Australia; ^2^ Foodbank WA Perth Airport WA Australia

**Keywords:** cooking, fast food, food insecurity, food literacy, food poverty, food security, takeaway

## Abstract

**Issue addressed:**

Vulnerable populations are disproportionately affected by food insecurity, resulting in heightened risk of suboptimal dietary intake. Food insecure people appear to implement several coping strategies and dietary compromises to avoid hunger. Less explored in the literature is how these strategies impact consumption of food inside and outside of the home.

**Methods:**

An online survey was completed by adults (n = 1292) residing in one of five Australian states. The questionnaire comprised of the six‐item US Household Food Security Survey Module, 12 socio‐demographic variables and 32 questions related to elements of food literacy.

**Results:**

Food insecure respondents were more likely to frequent fast food vs (*P* = .002), takeaway (*P* < .001) and food courts (*P* < .001) than their food secure counterparts. Food secure respondents reported greater use of raw (*P* = .043) and fresh, pre‐prepared produce (*P* = .002) when cooking, whereas food insecure respondents were more likely to prepare food using only frozen, pre‐packaged products (*P* < .001). No significant differences were found between food security status and the enjoyment and social bonding derived from cooking.

**Conclusions:**

Food insecure respondents appeared to be accessing a poorer quality of food through greater consumption of takeaway and fast food. These dietary compromises are most likely related to perceived financial, time or cooking facility constraints and to a lesser extent food literacy skills.

**So what?:**

This study highlights some of the health and social inequities apparent within food insecure populations. Food insecure households should be supported to access healthy fresh food and in‐home cooking practices. While a multi strategy approach is required, healthy food environment policy, particularly in disadvantaged areas, should be considered to guarantee that all Australians have dignified access to nutritious food.

## 1 INTRODUCTION

Food insecurity is a complex or ‘wicked problem representing a significant public health, economic and social policy concern.^1^ Food insecurity is apparent ‘whenever the availability of nutritionally adequate and safe foods, or the ability to acquire acceptable food in socially acceptable ways, is limited or uncertain’.^2^ The cultural boundaries of ‘socially acceptable’ in this study relate to the ability to purchase food from preferred retailers and prepare food that meets a person's health and social requirements without having to resort to theft or food relief.

Despite Australia producing twice as much food as it consumes,^3^ the country's population is not immune to food insecurity. Food insecurity is a divisive issue in Australia with ongoing debate around responsibility, contributing factors and potential solutions.^4^ Dialogue around food insecurity and hunger often emphasises personal responsibility, but this does not take into consideration the systematic drivers of the issue. Australia, similar to many other developed nations, has experienced stagnant wages and welfare payments for nearly two decades.^5^ These factors, coupled with an increasing cost of living, apply pressure to households and threaten food security with marginalised and disadvantaged groups the most vulnerable.^5^

Increasingly, economic and social shocks, such as job loss or divorce, have been implicated in decreased household resilience and capacity to ward off food insecurity. Despite this, food insecure households can be resourceful and appear to implement multiple strategies to avoid hunger.^6^ These coping strategies, however, may translate into differing consumption patterns exhibited by food insecure individuals when compared to the general population. Within the context of high‐income industrialised nations, several studies, investigating the dietary behaviours of food insecure populations, have cited reduced vegetable intake in conjunction with over consumption of high energy, nutrient‐poor foods and drinks.^7^, ^8^, ^9^, ^10^, ^11^, ^12^

Less explored within the literature are associations between food security status and elements of food literacy (such as home food preparation, cooking motivators and consumption of food outside of the home). Food literacy is defined as the ‘knowledge, skills and behaviours required to plan, manage, select, prepare and eat food to meet needs and determine food intake’.^13^ A study of adults residing in disadvantaged suburbs of Brisbane, Australia reported food insecurity was associated with more frequent hamburger consumption, but no other takeaway foods.^14^ A more significant relationship between food insecurity and takeaway intake was evident in participants of a Western Australian food literacy program.^7^ Lower self‐reported cooking skills and healthy food preparation in food insecure participants was another finding of the^7^ study. Similar results were cited by^11^ in low‐income African Americans residing in the US. In contrast, Canadian research found no difference in cooking abilities between food secure and insecure groups.^15^ Presently, there is no consensus whether a relationship between food security and cooking skills or dining out (including takeaway consumption) practices exists, while cooking motivators have not been explored.

To our knowledge, most Australian studies investigating cooking behaviours and dining out practices have been implemented within very specific food insecure subpopulations and none have been conducted across multiple Australian states. The objectives in this study were to examine if food security status was related to difference in^1^ dining out—selected frequency and type of ready to eat foods purchased, and^2^ dining in—food preparation behaviours and motivators in homes.

## 2 METHOD

### 2.1 Recruitment and sample population

Between November 2014 and February 2015 an online survey was administered to registered Australian panellists recruited through a commercial marketing research company. Panellists were emailed an invitation to participate in the survey titled ‘food shopping, food choice, cooking and consumption’ and a link to the online questionnaire. The online survey was constructed and data collected using Qualtrics (Provo, Utah, USA, 2014) software. The following inclusion criteria were applied: respondents must be adults (over 18 years of age), the primary household grocery shopper and reside in one of five Australian states (Western Australia, Victoria, Queensland, South Australia and New South Wales). Due to funding constraints and the capacity to get an adequate sample size, residents from the Northern Territory and the Australia Capital Territory were excluded from the sampling process. Respondents who did not meet the eligibility criteria were screened out. Access to the internet was necessary for completion of the online survey. Quotas for the number of respondents, age and location were established to ensure the study population was comparable to the general Australian population.^16^ In 2014, 14 million Australians^17^ were thought to be primary grocery shoppers and this estimate was used to calculate the sample required (n = 1024 respondents based on an Cohen's effect size of 0.20 (small), 5% level of significance and 80% power). A quota for the minimum number of survey completions was set at over 1000 responses; the survey link was emailed to the registered panellists until this quota was satisfied. Approximately one‐third of Australian primary household grocery shoppers are men and therefore adequate male representation was determined to be a minimum of 30%.^18^

### 2.2 Online survey development

An online survey was employed to investigate the study objectives. A mixed methods research design comprised of sequential steps was utilised to inform content and measurement scales included within the survey. Survey questions were initially generated from an extensive literature review and then tested using focus groups as recommended for good survey design. The function of these focus groups was to clarify language and determine appropriateness and relevance of the survey subject matter.^19^ Six focus groups were conducted, between September and October 2013, with primary grocery shoppers in Western Australia. Following the results of this qualitative stage, the survey content, scales and items were adjusted where necessary.

The finalised survey comprised of the six‐item US Household Food Security Survey Module (HFSSM), 12 socio‐demographic variables (including gender, age, occupation, household income, education, immigration, household structure and marital status) and 32 food literacy‐related questions (half related to food purchased or selected outside home and the other half about home food preparation and cooking questions).

These food literacy‐related questions explored aspects of the four components (select, prepare, plan and manage and eat) of food literacy.^13^ For the purpose of this research, the terms select or selection are used interchangeably and refer to access or purchase of food from a range of sources or locations. Prepare or preparation corresponds to the creation of meals from available ingredients. Plan and manage reflects the prioritisation of time and money spent on food. Eat encompasses cooking motivation and eludes to the ability of respondents use food in a social way.^13^ The design of these questions was informed by findings from the literature review and focus group analysis.^19^ In addition, the scale development for the dining out and cooking questions were influenced by previous research from.^20^

The USDA HFSSM^18^ has been extensively validated in the USA and has demonstrated capacity to accurately measure food insecurity.^21^ Within an Australian context, at least 11 peer reviewed studies have utilised a version of the HFSSM instrument to investigate food insecurity in various populations.^22^ The USDA HFSSM is the second most prolifically employed measure of food insecurity in Australia^22^; only behind the Australian Bureau of Statistics single item which has been criticised for underestimating the issue.^23^ Food security (FS) status in this study was determined using the six‐item HFSSM. The short form of this indicator was included, in favour of the longer version, due to the comparable accuracy (correctly identifies 97.7% food insecure households) and reduced respondent burden.^24^ In accordance with the HFSSM user notes.^25^ respondents’ FS status was defined as follows:

- *High‐Marginal FS* – Sufficient quantities of food with no changes to diet. Individuals with Marginal FS generally experience anxiety about potential food shortage, while those with High FS do not.
- *Low FS* – Food quantity is not affected, but variety, desirability and quality are reduced;
- *Very Low FS* – Decreased food intake of one or more household members and cyclical disruptions of diet.

The short form of the HFSSM is unable to distinguish between High and Marginal FS and for this reason these two groups were collapsed. Those experiencing Low or Very Low FS were considered to be food insecure for the purpose of this research.^25^ A more detailed methodology of the determination of FS status and the analysis of the associated socio‐demographic variables has been previously published.^26^

### 2.3 Statistics

Survey data was analysed using Statistical Package for Social Sciences (SPSS) (IBM Corp. Version 25). The four‐ to six‐point scale response categories for the food behaviours questions were reduced to a two‐ or three‐point scale when necessary due to low cell counts. The individual relationships between FS status, two socio‐demographic (household income and education) and the 32 food literacy questions were first explored via cross tabulations and chi‐square tests. All food literacy variables were then entered into a multinomial logistic regression model to formally examine their relationship with FS status. Four socio‐demographic factors (household income, education, age and marital status) were selected for inclusion as confounding factors in the model. These factors were chosen as they were significantly associated with FS status, as identified in our previous work investigating the relationship between multiple socio‐demographic variables and FS in two sample populations,^26^ and the same associations were observed in this study's data. Statistical significance was set at *P* ≤ .05.

## 3 RESULTS

### 3.1 Socio‐demographic characteristics

The survey was disseminated to 5431 Australians, with 1292 (23.8% response rate) completing all questions. The socio‐demographic features previously found to be significant independent predictors of FS status,^26^ and the gender ratio of the respondent population are outlined in Table 1. Approximately a third (35.2%) of the respondents were categorised as food insecure (Low FS 19.4%, n = 251 and Very Low FS 15.8%, n = 204). In terms of household income, respondents most frequently reported an income within the low (30.8%, n = 389) followed by the middle (24.1%, n = 311) and the high (23.6%, n = 305) brackets (Table 1). Respondents were predominately married or in de facto relationships (59.6%, n = 770) and just under three quarters (72.9%, n = 942) had completed postsecondary education.

**TABLE 1:** Characteristics of survey respondents compared by food security status

Independent variable	Category	Overall significance^a^	Total n (% of N = 1292)	High‐marginal food security n = 837 (64.8%)	Low food security n = 251 (19.4%)	Very low food security n = 204 (15.8%)
Gender		0.175				
	Male		603 (46.7%)	380 (45.4%)	131 (52.2%)	92 (45.1%)
	Female		689 (53.3%)	457 (54.6%)	120 (47.8%)	112 (54.9%)
Age (y)		<0.001**				
	19‐24		187 (14.5%)	88 (10.5%)	62 (24.7%)	37 (18.1%)
	25‐ 34		254 (19.7%)	142 (17.0%)	62 (24.7%)	50 (24.5%)
	35‐44		220 (17.0%)	119 (14.2%)	52 (20.7)	49 (0.24%)
	45‐54		226 (17.5%)	158 (18.9%)	35 (0.14%)	33 (16.2%)
	55‐64		210 (16.3%)	160 (19.1%)	30 (0.12%)	20 (9.8%)
	65‐84		195 (15.1%)	170 (20.3%)	10 (0.04%)	15 (7.4%)
Marital status		0.016**				
	Widowed		33 (2.6%)	28 (3.3%)	2 (0.8%)	3 (1.5%)
	Divorced/Separated		137 (10.6%)	85 (10.2%)	21 (8.4%)	31 (15.2%)
	Married/De facto		770 (59.6%)	528 (63.1%)	132 (52.6%)	110 (53.9%)
	Single		352 (27.2%)	196 (23.4%)	96 (38.2%)	60 (29.4%)
Household income ($AUD)		<0.001**				
	Very low (<$18,000)		40 (3.1%)	13 (1.6%)	15 (6.0%)	12 (5.9%)
	Low ($18,001‐37,000)		398 (30.8%)	241 (28.8%)	75 (29.9%)	82 (40.2%)
	Middle ($37,001‐87,000)		311 (24.1%)	202 (24.1%)	62 (24.7%)	47 (23.0%)
	High ($87,001‐180,000)		305 (23.6%)	212 (25.3%)	54 (21.5%)	39 (19.1%)
	Very high (>$180,000)		122 (9.4%)	86 (10.3%)	22 (8.8%)	14 (6.9%)
	Did not answer		116 (9.0%)	83 (9.9%)	23 (9.2%)	10 (4.9%)
Education completed		0.050*				
	Secondary or less		336 (26.0%)	206 (24.6%)	74 (29.5%)	56 (27.5%)
	Vocational^b^		515 (39.9%)	328 (39.2%)	96 (38.2%)	91 (44.6%)
	University		427 (33.0%)	291 (34.8%)	81 (32.3%)	55 (27.0%)

^a^ Multinomial logistic regression was used to establish significance.

^b^ Vocational considered to be postsecondary.

* *p* ≤ .05;

** *p* ≤ .01.

### 3.2 Dining out

Table 2 outlines the relationship between 16 dining out variables and FS status. Response rates to the dining out questions are available in Table S1.

**TABLE 2:** Response, by three categories of food security p‐values and odds ratios of dining out questions

Outcome	Category	Overall significance^a^	Post hoc analysis					
			High‐marginal vs very low food security		High‐marginal vs low food security		Low food security vs very low food security	
			OR (95% CI)	*p*‐value	OR (95% CI)	*p*‐value	OR (95% CI)	*p*‐value
In an average month, how often do you dine out at the following?								
Full service restaurant		0.442						
	Less than once a month		1.00 (ref)		1.00 (ref)		1.00 (ref)	
	1‐2 times		1.34 (0.86, 2.08)	.195	1.32 (0.89, 1.96)	.171	1.01 (0.61, 1.70)	.958
	3‐5 times		0.95 (0.51, 1.77)	.865	0.78 (0.46, 1.32)	.346	1.22 (0.61, 2.43)	.567
	>6 times		0.61 (0.23, 1.60)	.316	0.95 (0.35, 2.56)	.917	0.64 (0.21, 2.02)	.451
Cafe		0.044*						
	Less than once a month		1.00 (ref)		1.00 (ref)		1.00 (ref)	
	1‐2 times		0.77 (0.52, 1.13)	.183	0.58 (0.41, 0.83)	.003**	1.33 (0.85, 2.07)	.212
	3‐5 times		0.84 (0.51, 1.38)	.492	0.71 (0.45, 1.13)	.146	1.18 (0.66, 2.09)	.577
	> 6 times		0.54 (0.27, 1.07)	.079	0.46 (0.24, 0.88)	.018*	1.17 (0.54, 2.53)	.697
Takeaway (eat at the location)								
	Less than once a month	<0.001**	1.00 (ref)		1.00 (ref)		1.00 (ref)	
	1‐2 times		0.54 (0.36, 0.81)	.003**	0.47 (0.32, 0.67)	<.001**	1.16 (0.74, 1.81)	.514
	3‐5 times		0.46 (0.24, 0.86)	.016*	0.41 (0.23, 0.72)	.002**	1.11 (0.57, 2.18)	.758
	>6 times		0.37 (0.15, 0.89)	.027*	0.44 (0.19, 1.01)	.053	0.84 (0.33, 2.12)	.712
Takeaway (eat at the home)								
	Less than once a month	0.031*	1.00 (ref)		1.00 (ref)		1.00 (ref)	
	1‐2 times		0.96 (0.65, 1.43)	.855	0.64 (0.45, 0.90)	.011*	1.52 (0.97, 2.37)	.069
	3‐5 times		0.87 (0.53, 1.45)	.599	0.78 (0.48, 1.26)	.306	1.12 (0.62, 2.00)	.706
	>6 times		0.51 (0.23, 1.14)	.102	0.32 (0.16, 0.66)	.002**	1.56 (0.70, 3.47)	.273
Fast food (eat at the location)		0.002**						
	Less than once a month		1.00 (ref)		1.00 (ref)		1.00 (ref)	
	1‐2 times		0.80 (0.54, 1.19)	.270	0.46 (0.33, 0.66)	<.001**	1.73 (1.11, 2.68)	.015*
	3‐5 times		0.81 (0.48, 1.38)	.439	0.69 (0.42, 1.14)	.149	1.17 (0.64, 2.15)	.612
	>6 times		0.74 (0.31, 1.75)	.489	0.44 (0.21, 0.90)	.024*	1.69 (0.70, 4.09)	.243
In an average month, how often do you dine out at the following?								
Fast food (eat at the home)		0.076						
	Less than once a month		1.00 (ref)		1.00 (ref)		1.00 (ref)	
	1‐2 times		0.88 (0.60, 1.30)	.525	0.69 (0.48, 1.00)	.048*	1.27 (0.81, 2.01)	.297
	3‐5 times		1.09 (0.67, 1.76)	.731	0.69 (0.45, 1.06)	.088	1.58 (0.91, 2.74)	.101
	>6 times		0.75 (0.36, 1.60)	.462	0.38 (0.20, 0.72)	.003**	1.97 (0.92, 4.23)	.083
Food court		<0.001**						
	Less than once a month		1.00 (ref)		1.00 (ref)		1.00 (ref)	
	1‐2 times		0.70 (0.48, 1.04)	.077	0.56 (0.39, 0.80)	.002**	1.26 (0.79, 1.99)	.335
	3‐5 times		0.37 (0.23, 0.59)	<.001**	0.31 (0.20, 0.48)	<.001**	1.19 (0.70, 2.01)	.517
	> 6 times		0.30 (0.16, 0.57)	<.001**	0.34 (0.19, 0.62)	<.001**	0.89 (0.45, 1.77)	.736
Please indicate the level of importance for the following features of a food court:								
Convenient location		<0.001**						
	Important		1.00 (ref)		1.00 (ref)		1.00 (ref)	
	Neither important nor unimportant		0.10 (0.03, 0.37)	.001**	0.09 (0.02, 0.31)	<.001**	1.14 (0.43, 3.05)	.798
	Unimportant		0.58 (0.31, 1.08)	.467	0.46 (0.27, 0.78)	.004**	1.27 (0.67, 2.38)	.467
Cultural familiarity with food options		0.263						
	Important		1.00 (ref)		1.00 (ref)		1.00 (ref)	1.00 (ref)
	Neither important nor unimportant		1.28 (0.68, 2.42)	.441	0.91 (0.49, 1.71)	.772	1.41 (0.67, 2.96)	.366
	Unimportant		1.07 (0.57, 1.99)	.844	1.61 (0.87, 2.99)	.131	1.72 (0.82, 3.54)	.144
Affordability		0.055						
	Important		1.00 (ref)		1.00 (ref)		1.00 (ref)	
	Neither important nor unimportant		0.92 (0.25, 3.29)	.892	1.19 (0.31, 4.62)	.798	0.77 (0.16, 3.65)	.738
	Unimportant		1.07 (0.52, 2.21)	.846	0.47 (0.27, 0.80)	.006**	2.30 (1.12, 4.75)	.024*
Please indicate the level of importance for the following features of a food court.								
Speed of service		0.026*						
	Important		1.00 (ref)		1.00 (ref)		1.00 (ref)	
	Neither important nor unimportant		0.30 (0.09, 0.96)	.043*	0.45 (0.13, 1.51)	.194	0.66 (0.19, 2.32)	.519
	Unimportant		1.00 (0.54, 1.85)	.988	0.54 (0.33, 0.87)	.012*	1.86 (0.98, 3.52)	.058
Whatever is most convenient		0.199						
	Important		1.00 (ref)		1.00 (ref)		1.00 (ref)	1.00 (ref)
	Neither important nor unimportant		2.48 (1.11, 5.58)	.100	1.80 (0.85, 3.80)	.125	1.38 (0.59, 3.26)	.460
	Unimportant		1.70 (0.78, 3.71)	.184	0.57 (0.27, 1.19)	.134	0.96 (0.44, 2.20)	.930
Something inexpensive		0.728						
	Important		1.00 (ref)		1.00 (ref)		1.00 (ref)	
	Neither important nor unimportant		0.94 (0.42, 2.12)	.883	1.32 (0.56, 3.10)	.523	0.71 (0.26, 1.92)	.504
	Unimportant		1.29 (0.78, 2.13)	.327	0.96 (0.63, 1.47)	.844	1.34 (0.77, 2.34)	.298
Value for money		0.005**						
	Important		1.00 (ref)		1.00 (ref)		1.00 (ref)	
	Neither important nor unimportant		0.24 (0.07, 0.84)	.025*	0.31 (0.09, 1.01)	.051	0.80 (0.26, 2.51)	.703
	Unimportant		1.20 (0.60, 2.38)	.605	0.50 (0.30, 0.84)	.009**	2.40 (1.19, 4.83)	.015*
Recognised brand		0.839						
	Important		1.00 (ref)		1.00 (ref)		1.00 (ref)	1.00 (ref)
	Neither important nor unimportant		1.16 (0.58, 2.32)	.680	0.57 (0.26, 1.23)	.149	1.59 (0.70, 3.62)	.271
	Unimportant		0.86 (0.44, 1.67)	.658	1.22 (0.63, 2.34)	.549	0.70 (0.32, 1.56)	.386
On average, how much do you spend per person on one trip to a food court?		<0.001**						
	$0‐10		1.00 (ref)		1.00 (ref)		1.00 (ref)	
	$11‐20		0.94 (0.58, 1.51)	.784	0.74 (0.48, 1.14)	.168	1.27 (0.73, 2.22)	.399
	$21‐30		0.38 (0.18, 0.78)	.008**	0.29 (0.15, 0.58)	<.001**	1.28 (0.59, 2.76)	.531
	>$31		0.17 (0.06, 0.49)	.001**	0.15 (0.06, 0.40)	<.001**	1.15 (0.46, 2.89)	.760

Abbreviations: 1.00 (ref), reference level; CI, confidence interval; OR, odds ratio.

^a^ Multinomial logistic regression model was adjusted for socio‐demographic variables (age, household income, education and marital status).

* *P* ≤ .05;

** *P* ≤ .01.

### 3.3 Food selection – point of purchase and location

The majority of the location and food type questions (five of seven) were significantly related to FS status (Table 2). On average and in the last month, food insecure respondents were more likely to frequent cafes (*P* = .044), fast food venues (food eaten at the location, *P* = .002), takeaway (food eaten at the location, *P* < .001 and food eaten at home, *P* = .031) and food courts (*P* < .001) than their food secure counterparts. In relation to the fast food and takeaway items, the association between frequencies of use by food insecure respondents was greater when food was consumed at the venue. Significant disparities in reported number of visits to dining out facilities were most apparent between the Low FS, greatest frequency and High‐Marginal FS, lowest frequency, groups.

### 3.4 Planning and management – food court features

When asked to rate the importance of eight food court features, only three were found to have a significant association with FS status (Table 2). Food insecure respondents were the more likely to rate speed of service (*P* = .026), value for money (*P* = .005) and convenient location (*P* < .001) as important characteristics of food courts compared with High‐Marginal FS respondents. No significant difference between FS status was observed between recognised brand, something inexpensive, whatever is most convenient, affordability or cultural familiarity with food options, however, food insecure respondents were more likely to report spending more money per person on each trip to food courts (*P* < .001) than their food secure counterparts.

When adjusted for the socio‐demographic variables, over half (n = 9) of the 16 dining out indicators assessed were significantly associated with FS status (refer to Table 2). Of these, five were related to food location and type, three referred to features of food courts and one referenced the average amount of money spent per food court visit.

### 3.5 Dining in

Table 3 describes the relationship between six food preparation and 10 cooking motivator variables and FS status. Socio‐demographic characteristics have been controlled for in each analysis. Table S2 provides the response rates to the dining in items.

**TABLE 3:** Response, by three categories of food security p‐values and odds ratios of dining in questions

Outcome	Category	Overall significance^a^	Post hoc analysis					
			High‐marginal vs very low food security		High‐marginal vs low food security		Low food security vs very low food security	
			OR (95% CI)	*p*‐value	OR (95% CI)	*p*‐value	OR (95% CI)	*p*‐value
How healthy is your diet?		0.050*						
	Healthy		1.58 (1.09, 2.29)	.016*	1.22 (0.86, 1.73)	.260	1.29 (0.85, 1.97)	.231
	Unhealthy		1.00 (ref)		1.00 (ref)		1.00 (ref)	
In an average week, how many dinner time meals do you cook?								
Using all raw produce for example, fresh vegetables, unprepared meats		0.043*						
	Rarely		1.00 (ref)		1.00 (ref)		1.00 (ref)	
	Less than once		0.48 (0.23, 0.98)	.044*	0.40 (0.20, 0.79)	.008**	1.21 (0.54, 2.73)	.644
	1‐2 times		0.64 (0.35, 1.14)	.131	0.49 (0.29, 0.87)	.015*	1.29 (0.65, 2.57)	.466
	3‐4 times		1.01 (0.57, 1.79)	.977	0.68 (0.39, 1.19)	.180	1.47 (0.74, 2.93)	.268
	5‐6 times		1.09 (0.60, 1.98)	.788	0.82 (0.46, 1.47)	.499	1.32 (0.64, 2.74)	.442
	Everyday		1.28 (0.65, 2.53)	.479	0.73 (0.39, 1.37)	.323	1.76 (0.79 3.95)	.169
Using a mixture of pre‐packaged and fresh (raw) produce for example, fresh meat, spaghetti and a can/bottle sauce		0.509						
	Rarely		1.00 (ref)		1.00 (ref)		1.00 (ref)	
	Less than once		0.71 (0.37, 1.36)	.306	0.77 (0.41, 1.42)	.394	0.93 (0.43, 2.03)	.858
	1‐2 times		0.67 (0.38, 1.17)	.159	0.66 (0.39, 1.11)	.118	1.01 (0.52, 1.20)	.971
	3‐4 times		0.55 (0.30, 1.01)	.055	0.63 (0.36, 1.12)	.115	0.87 (0.42, 1.80)	.711
	5‐6 times		0.92 (0.41, 2.09)	.842	0.58 (0.29, 1.15)	.120	1.59 (0.62, 4.05)	.328
	Everyday		1.04 (0.38, 2.84)	.934	1.28 (0.48, 3.37)	.623	0.89 (0.24, 2.78)	.747
Using only frozen pre‐packaged products for example, only defrosting and heating required		<0.001**						
	Rarely		1.00 (ref)		1.00 (ref)		1.00 (ref)	
	Less than once		0.86 (0.56, 1.34)	.520	0.64 (0.43, 0.94)	.024*	1.36 (0.81, 2.29)	.244
	1‐2 times		0.43 (0.28, 0.66)	<.001**	0.39 (0.26, 0.58)	<.001**	1.12 (0.68, 1.84)	.655
	3‐4 times		0.38 (0.21, 0.69)	.002**	0.33 (0.19, 0.57)	<.001**	1.18 (0.62, 2.26)	.618
	5‐6 times		0.45 (0.17, 1.19)	.107	0.30 (0.13, 0.71)	.006**	1.49 (0.53, 4.19)	.447
	Everyday		0.61 (0.20, 1.90)	.394	0.64 (0.20, 2.06)	.456	0.95 (0.26, 3.42)	.934
In an average week, how many dinner time meals do you cook?								
Using fresh, but pre‐prepared produce for example, meat with spices or marinade or meat‐filled pasta from the refrigerated section		0.002**						
	Rarely		1.00 (ref)		1.00 (ref)		1.00 (ref)	
	Less than once		0.77 (0.51, 1.18)	.228	0.69 (0.46, 1.04)	.074	1.11 (0.67, 1.85)	.681
	1‐2 times		0.70 (0.45, 1.10)	.127	0.53 (0.35, 0.81)	.003**	1.32 (0.78, 2.23)	.301
	3‐4 times		0.42 (0.23, 0.76)	.004**	0.30 (0.17, 0.51)	<.001**	1.42 (0.75, 2.68)	.277
	5‐6 times		0.45 (0.18, 1.13)	.090	0.40 (0.17, 0.96)	.041*	1.12 (0.40, 3.14)	.832
	Everyday		0.92 (0.26, 3.28)	.894	0.48 (0.16, 1.39)	.176	1.92 (0.51, 7.24)	.333
I make more of an effort when cooking for								
Celebrations/Anniversary		0.042*						
	Never		1.00 (ref)		1.00 (ref)		1.00 (ref)	
	Rarely		0.42 (0.22, 0.82)	.010**	0.95 (0.49, 1.85)	.870	0.45 (0.20, 0.97)	.043*
	Sometimes		0.69 (0.38, 1.24)	.214	0.70 (0.41, 1.18)	.181	0.98 (0.50, 1.93)	.957
	Often		0.95 (0.51, 1.76)	.872	0.86 (0.50, 1.46)	.573	1.11 (0.55, 2.22)	.772
	Always		0.85 (0.46, 1.59)	.617	1.04 (0.59, 1.81)	.898	0.82 (0.40, 1.69)	.596
Guests		0.047*						
	Never		1.00 (ref)		1.00 (ref)		1.00 (ref)	
	Rarely		0.49 (0.22, 1.04)	.063	0.57 (0.28, 1.19)	.133	0.83 (0.34, 2.03)	.688
	Sometimes		0.49 (0.25, 0.96)	.037*	0.57 (0.31, 1.05)	.071	0.86 (0.40, 1.87)	.710
	Often		0.75 (0.38, 1.49)	.416	0.65 (0.35, 1.19)	.160	1.17 (0.53, 2.57)	.700
	Always		0.83 (0.42, 1.64)	.586	0.97 (0.52, 1.84)	.931	0.85 (0.38, 1.91)	.694
There are other occasions that I make more of an effort when cooking		0.003**						
	Agree		1.00 (ref)		1.00 (ref)		1.00 (ref)	
	Disagree		0.61 (0.44, 0.84)	0.002**	0.69 (0.52, 0.94)	.016*	0.87 (0.60, 1.27)	.475
Rate your level of agreement								
I cook for the sake of eating		0.138						
	Strongly disagree		1.00 (ref)		1.00 (ref)		1.00 (ref)	
	Disagree		1.78 (0.89, 3.55)	.101	0.94 (0.50, 1.76)	.846	1.89 (0.85, 4.24)	.120
	Neither agree nor disagree		1.02 (0.54, 1.92)	.953	0.95 (0.51, 1.75)	.862	1.08 (0.51, 2.30)	.849
	Agree		0.90 (0.48, 1.67)	.736	0.88 (0.48, 1.62)	.691	1.02 (0.48, 2.13)	.967
	Strongly agree		0.87 (0.40, 1.89)	.718	1.40 (0.62, 3.20)	.421	0.62 (0.24, 1.62)	.328
I feel cooking is a core		0.340						
	Strongly disagree		1.00 (ref)		1.00 (ref)		1.00 (ref)	
	Disagree		0.95 (0.47, 1.93)	.878	0.89 (0.44, 1.78)	.736	1.07 (0.44, 2.57)	.886
	Neither agree nor disagree		0.67 (0.34, 1.33)	.254	0.55 (0.28, 1.08)	.084	1.21 (0.52, 2.80)	.655
	Agree		0.69 (0.34, 1.38)	.291	0.66 (0.33, 1.31)	.234	1.039 (0.44, 2.455)	.930
	Strongly agree		0.64 (0.28, 1.49)	.303	0.70 (0.30, 1.62)	.400	0.92 (0.33, 2.57)	.880
Cooking is an opportunity to bond with my family		0.496						
	Disagree		1.00 (ref)		1.00 (ref)		1.00 (ref)	
	Neither agree nor disagree		0.95 (0.60, 1.51)	.825	0.94 (0.62, 1.42)	.753	1.02 (0.60, 1.73)	.957
	Agree		0.61 (0.31, 1.19)	.143	0.60 (0.32, 1.11)	.103	1.01 (0.48, 2.13)	.976
Cooking is an opportunity to bond with my friends		0.145						
	Strongly disagree		1.00 (ref)		1.00 (ref)		1.00 (ref)	
	Disagree		0.56 (0.26, 1.23)	.151	0.72 (0.32, 1.62)	.431	0.78 (0.29, 2.07)	.617
	Neither agree nor disagree		0.94 (0.45, 2.00)	.881	0.64 (0.30, 1.36)	.236	1.49 (0.59, 3.76)	.404
	Agree		0.61 (0.29, 1.28)	.187	0.52 (0.24, 1.10)	.085	1.17 (0.47, 2.95)	.738
	Strongly agree		0.85 (0.32, 2.22)	.737	0.73 (0.28, 1.89)	.510	1.17 (0.36, 3.79)	.795
Cooking is important to me because I know exactly what I am eating		0.923						
	Disagree		1.00 (ref)		1.00 (ref)		1.00 (ref)	
	Neither agree nor disagree		1.12 (0.63, 2.00)	.693	1.07 (0.64, 1.80)	.792	1.05 (0.55, 2.00)	.888
	Agree		1.08 (0.64, 1.83)	.775	1.20 (0.74, 1.94)	.468	0.90 (0.50, 1.63)	.736
Rate your level of agreement								
I enjoy cooking		0.746						
	Strongly disagree		1.00 (ref)		1.00 (ref)		1.00 (ref)	
	Disagree		1.10 (0.50, 2.40)	.817	0.85 (0.38, 1.87)	.679	1.30 (0.50, 3.38)	.594
	Neither agree nor disagree		1.12 (0.54, 2.31)	.770	0.64 (0.30, 1.33)	.228	1.75 (0.72, 4.27)	.217
	Agree		1.03 (0.51, 2.08)	0.932	0.88 (0.42, 1.82)	.728	1.17 (0.49, 2.81)	.720
	Strongly agree		0.94 (0.44, 2.04)	.880	0.80 (0.36, 1.77)	.579	1.18 (0.46, 3.04)	.732
Cooking makes me feel good		0.892						
	Strongly disagree		1.00 (ref)		1.00 (ref)		1.00 (ref)	
	Disagree		0.98 (0.43, 2.22)	.952	0.78 (0.34, 1.83)	.570	1.25 (0.46, 3.41)	.667
	Neither agree nor disagree		1.16 (0.55, 2.46)	.703	0.67 (0.31, 1.44)	.301	1.74 (0.69, 4.36)	.239
	Agree		0.93 (0.44, 1.93)	.835	0.76 (0.35, 1.64)	.481	1.22 (0.49, 3.03)	.669
	Strongly agree		1.08 (0.47, 2.48)	.855	0.72 (0.31, 1.68)	.453	1.49 (0.55, 4.09)	.436
On average, how much time do you spend on preparing your dinner meal?								
Weekends		0.074						
	15 minutes or less		1.00 (ref)		1.00 (ref)		1.00 (ref)	
	15‐30 minutes		0.70 (0.39, 1.27)	.245	0.58 (0.33, 1.03)	.064	1.21 (0.56, 2.46)	.610
	30‐45 minutes		0.62 (0.34, 1.13)	.117	0.48 (0.27, 0.85)	.012*	1.29 (0.63, 2.63)	.482
	45 ‐ 60 minutes		0.85 (0.44, 1.64)	.635	0.89 (0.47, 1.69)	.728	0.96 (0.43, 2.14)	.913
	60 < minutes		0.94 (0.42, 2.13)	.885	0.46 (0.23, 0.93)	.030*	2.06 (0.81, 5.28)	.131
Week days		0.075						
	15 minutes or less		1.00 (ref)		1.00 (ref)		1.00 (ref)	
	15‐30 minutes		0.93 (0.52, 1.66)	.802	0.64 (0.36, 1.15)	.135	1.44 (0.71, 2.95)	.315
	30‐45 minutes		0.68 (0.38, 1.20)	.182	0.42 (0.24, 0.75)	.003**	1.60 (0.79, 3.23)	.191
	45 ‐ 60 minutes		0.80 (0.42, 1.51)	.484	0.70 (0.37, 1.34)	.284	1.13 (0.52, 2.49)	.757
	60 < minutes		0.96 (0.40, 2.32)	.925	0.51 (0.23, 1.13)	.097	1.88 (0.68, 5.22)	.225

Abbreviations: 1.00 (ref), reference level; CI, confidence interval; OR, odds ratio.

^a^ Multinomial logistic regression model was adjusted for socio‐demographic variables (age, household income, education and marital status).

* *p* ≤ .05;

** *p* ≤ .01.

### 3.6 Food preparation, planning and management

There was no significant difference among all three respondent FS groups in terms of time required to prepare meals both during the week (*P* = .075) and on the weekend (*P* = .074), however, significant associations were observed with regard to the types of products used for cooking. High‐Marginal FS respondents reported more frequent use of all raw produce (*P* = .043) and using fresh, but pre‐prepared produce (*P* = .002) when cooking, whereas the food insecure participants were more likely to cite using only frozen pre‐packaged products to prepare meals (*P* < .001) during the week. When asked to rate how healthy their diet was (Table 3), High‐Marginal FS respondents were one and a half times more likely to describe their diet as healthy than Very Low FS respondents (*P* = .016).

### 3.7 Eat‐cooking motivators

Food insecure respondents were less likely than food secure respondents to report making more of an effort when cooking for celebrations and anniversaries (*P* = .042), guests (*P* = .047) and other special occasions (*P* = .003). There were no differences between FS status and the other cooking motivators.

## 4 DISCUSSION

Our study provides an insight into the dining behaviours of a food insecure subpopulation within a large national sample, both in terms of food eaten outside home and food prepared at the residence. This evidence adds to the current Australian knowledge base by highlighting the potential social and dietary compromises made by food insecure people to minimise the impact of adversity.

The participants in this study comprised of grocery shoppers who also needed to be fluent in written English, with internet access. The sample was designed to be representative of the general population, with quotas for gender, age and location. This was done in order to able to compare those who were food secure within this group to those who were not. The sample does not reflect an impoverished population typically associated with food insecurity, and over half of our participants had a middle‐ or high‐income level. While a higher income is considered a protective factor and may afford greater resilience to shocks or stressors, it cannot be viewed as proxy for food security, as income does not necessarily reflect the disposable income or economic resourcing within a household.^27^ Higher income households are not immune to food insecurity when household expenses are significant or income is unexpectedly lost.^28^, ^29^

### 4.1 Dining out

Home cooking is promoted by the public health sector as a means of improving dietary intake and health outcomes.^30^ However, over the last couple of decades in Australia there has been a rise in convenience culture and a trend to prepare fewer meals at home.^31^ Similarly to other developed nations, this shift in dietary patterns has been coupled with increasing rates of overweight, obesity and associated chronic disease.^32^ Socially and economically disadvantaged groups, including people experiencing food insecurity, appear to be particularly vulnerable and are specially targeted by fast or convenience food marketing.^33^ Indeed, the greater density of fast food and takeaway outlets apparent in lower socio‐economic areas is evidenced in strategic targeting.^33^, ^34^ With this in mind, food insecure respondents in our study were potentially more likely to reside in areas where fast food outlets were easily accessible compared to their food secure counterparts. Fast food and takeaway outlets may represent a convenient and inexpensive option for food insecure people who may have time, financial and social constraints. Another consideration reported in the literature suggests individuals who are experiencing food insecurity are theorised to choose food with high caloric value instead of foods with high nutritional value as a biological means of achieving energy requirements for the least financial investment.^35^, ^36^, ^37^ Fast food and other takeaway options can fulfil this requirement through the provision of energy dense food without the need for kitchen facilities or cooking skills.^38^

A unique finding from our research is food insecure respondent's preference to eat at the food venue rather than taking purchased items home. Previous research has indicated that food insecure people rely more on public transport,^39^ are less likely to own a vehicle^40^ and have greater likelihood of residing in shared accommodation.^41^ Fast food and takeaway venues may represent a convenient, readily available and safe location to consume food for those who are food insecure. More research is warranted to explore these themes further.

Our findings suggest that food insecure respondents were more likely to consider *speed of service* and *convenience* as important aspects of dining out facilities when compared to their food secure counterparts. Another distinction that should be noted were participants experiencing food insecurity were not more interested in *something inexpensive* or the cheapest option, but rather perceived the food or meal to represent *value for money*. Aside from these aspects, customers of dining out facilities in our study appeared to favour similar features, such as *recognised brand* and *cultural familiarity*, regardless of their FS status. Large fast food company brand recognition has previously been found to be universal (regardless of age, gender and income)^42^ and this could explain why no significant difference was apparent between respondent groups in our study.

### 4.2 Dining in

When compared to FS respondents, participants experiencing food insecurity in this study cited less frequent use of raw unprocessed foods instead favouring frozen and prepared products. It is likely the evident variation in type of foods used by food insecure respondents for cooking may be a mechanism employed to keep spending to a minimum. Indeed, a reduction in variety and quality of food^43^, ^44^, as well as, a preference for convenience items^45^, ^46^ are coping strategies previously found to be associated with food insecurity. Frozen and canned foods tend to be cost effective, potentially time saving products with extended shelf lives, frequently provided as part of emergency food relief,^47^ and therefore are attractive to disadvantaged groups with restricted budgets.^48^ An important aspect to note is that canned or frozen products are not necessarily nutritionally inferior^49^ Low‐income women have been found to invest in frozen vegetables as a means of providing their households nutrient dense food for reduced cost, which is unaffected by seasonality.^50^

The impact of food literacy on food security status is controversial in the literature. Food literate individuals may be able to more effectively budget and meal plan, ameliorating the household's ability to withstand mild stressors and building resilience to food insecurity.^7^, ^51^ Lower cooking self‐efficacy ratings in food insecure groups have been reported in previous studies,^7^, ^52^, ^53^ so greater reliance on prepared foods or convenience foods (frozen and canned) could also be symptomatic of limited cooking skills. However, simplistic meal preparation exhibited by food insecure respondents could be related more to ownership of fewer cooking appliances or access to suitable facilities rather than skill level.^54^ There is a growing body of evidence, particularly from North America, citing food insecure people are very resourceful implementing many coping strategies to lessen the impact of the issue on food intake.^6^, ^15^, ^55^ Food insecure carers in the USA were found to be very skilful at stretching meals, making substitutes and cooking nontraditional meals.^6^ These aforementioned skills are indicative of a high level of food literacy. In support of the notion that reliance on prepared foods may not be entirely related to food literacy knowledge, we found no significant difference in the time spent on meal preparation between the food secure and insecure groups. However, other research suggests that food insecure people tend to appear to have lower food literacy due to financial constraints, which may inhibit their ability to practice food literacy skills (for example meal planning and budgeting).^15^, ^55^, ^56^, ^57^

Despite potentially implementing several coping strategies, food insecure respondents in our study were more likely to self‐assess their diet as unhealthy compared to those who were food secure. Respondents with Very Low FS were least likely to identify their diet as healthy, indicating that diet quality deteriorates with increasing food insecurity. The lack of consensus within the literature highlights the intricate and multifaceted relationship between food insecurity, food literacy and poor diet quality. Ultimately, plethora of factors are likely to affect an individual's food purchasing decision process. Although the individual has the capacity to change some of these factors (eg food literacy skills), political, economic and social determinants must be addressed at systems and organisational level to affect this complex problem.

Our research found that the FS status was not an associated factor for reported enjoyment and social bonding derived from cooking and dining together. In contrast to this, US research found individuals residing in a high‐income neighbourhood were more likely to view cooking as a hobby and derive pleasure from the activity than people living in a low‐income neighbourhood.^58^ Enjoyment was perceived to be a cooking facilitator, in the same US study, by all respondents; however, low‐income participants reported time and the affordability of ingredients as barriers to participation.^58^ It should be noted that preparation time and cooking motivators are not direct measures of cooking abilities or skills.

Food insecure respondents were less likely to report *making a greater effort when cooking for guests* and for *special occasions*. Food insecure people may feel some social exclusion due to limited finances, resulting in the inability to purchase special celebratory food.^59^ In light of this, it seems logical that food insecure respondents made less of an effort for special occasions and those experiencing the severest form of food insecurity (Very Low FS) were the least equipped to cater for special occasions or welcome guests for meals. When taken into consideration both food secure and insecure respondents in our study reported similar enjoyment and social bonding derived from cooking, Very Low FS respondents appeared to have less opportunity to enjoy these special occasions with friends or family at home.

Several limitations should be considered when interpreting these findings. This study investigates the food choice behaviours of Australians who respond to digital surveys, which represents only one of many types of food insecurity that is, those with access to the internet residing in a postindustrial democracy. Despite employing demographic quotas, our study population was similar to, but not a representative sample of the general Australian population. Respondents were not included from the Northern and Australian Captital territories; additionally when compared to the general population there was an overrepresentation of survey participants in the very high‐income bracket and in the postsecondary attainment category. Two important social determinants of FS in Australia that were not investigated specifically in this research were geographical isolation and Aboriginal and Torres Strait Islander status.^60^, ^61^ All survey data were self‐reported and derived from a convenience sample. Therefore results are subject to social desirability and sampling bias.^62^ The short form of the HFSSM is unable to distingush between High and Marginal FS. There is growing body of evidence about the detrimental affects of anxiety on food aquision experienced by those with Maringal FS and this translated to differing food behaviors, suggesting that High and Marginal FS groups should be considered seperately.

Additionally, only a few elements of food literacy excluding nutrition knowledge, budgeting skills and cooking abilities were assessed in this study meaning comprehensive conculsions about the area cannot be drawn. Our findings should be viewed as making a contribution to the understanding of how food insecurity may impact on elements of food literacy behaviour, but generalising these results to all groups is cautioned.

## 5 CONCLUSION

Our study found, regardless of food security status, respondents derived enjoyment and social bonding from cooking. A point of difference was that food insecure respondents in our study appeared to be accessing a poorer quality of food in terms of both selection and preparation. This study provides insight into the prevailing food choice behaviours of food insecure respondents, further highlighting the social and health inequities apparent within disadvantaged groups. The presence of food insecurity and the resultant dietary compromises can be viewed as a failure of political, social and economic systems.^63^ Australia currently has no national nutrition or food security policies. Investment in these policy areas and support for healthy food environments could potentially enhance the health outcomes of food insecure people by facilitating access to an improved quality of food.

## CONFLICT OF INTEREST

The authors declare no conflict of interest.

## ETHICS APPROVAL STATEMENT

All procedures performed in studies involving human participants were in accordance with the ethical standards of the institutional and/or national research committee and with the 1964 Helsinki declaration and its later amendments or comparable ethical standards. Informed consent was obtained from all individual participants included in the study. Ethics approval has been granted by Edith Cowan University's Human Research Ethics Committee.

## Supporting information

Table S1
