# Supplementary material for: To dine in or not to dine in: A comparison of food selection and preparation behaviours in those with and without food security
Source: Health Promot J Austr. 2020 Oct 20;32(Suppl 2):267–82. doi: 10.1002/hpja.427 (PMC8597035; doi:10.1002/hpja.427)
Supplement: Supplementary file 1 — Table S1 [file HPJA-32-267-s001.docx]

| **Outcome** | **Category** | **High-Marginal Food Security** | **Low Food Security** | **Very Low Food Security** |
| --- | --- | --- | --- | --- |
| **In an average month, how often do you dine out at the following?** |  |  |  |  |
| Full service restaurant | Less than once a month | 573 (68%) | 170 (68%) | 146 (72%) |
|  | 1-2 times | 189 (23%) | 48 (19%) | 35 (17%) |
|  | 3-5 times | 57 (7%) | 27 (11%) | 16 (8%) |
|  | > 6 times | 18 (2%) | 6 (2%) | 7 (3%) |
| Cafe | Less than once a month | 422 (50%) | 96 (38%) | 96 (47%) |
|  | 1-2 times | 251 (30%) | 96 (38%) | 64 (31%) |
|  | 3-5 times | 124 (15%) | 41 (17%) | 30 (15%) |
|  | > 6 times | 40 (5%) | 18 (7%) | 14 (7%) |
| Take away (eat at the location) | Less than once a month | 634 (76%) | 140 (56%) | 125 (61%) |
|  | 1-2 times | 147 (17%) | 72 (29%) | 51 (25%) |
|  | 3-5 times | 39 (5%) | 27 (11%) | 18 (9%) |
|  | > 6 times | 17 (2%) | 12 (5%) | 10 (5%) |
| Take away (eat at the home) | Less than once a month | 486 (58%) | 105 (42%) | 107 (53%) |
|  | 1-2 times | 234 (28%) | 91 (36%) | 57 (28%) |
|  | 3-5 times | 98 (12%) | 33 (13%) | 28 (14%) |
|  | > 6 times | 19 (2%) | 22 (9%) | 12 (6%) |
| Fast food (eat at the location) | Less than once a month | 559 (67%) | 112 (45%) | 117 (57%) |
|  | 1-2 times | 178 (21%) | 91 (36%) | 53 (27%) |
|  | 3-5 times | 78 (9%) | 30 (12%) | 25 (12%) |
|  | > 6 times | 22 (3%) | 18 (7%) | 9 (4%) |
| Fast food (eat at the home) | Less than once a month | 459 (55%) | 90 (36%) | 92 (45%) |
|  | 1-2 times | 227 (27%) | 81 (32%) | 64 (32%) |
|  | 3-5 times | 123 (15%) | 53 (21%) | 35 (17%) |
|  | > 6 times | 28 (3%) | 27 (11%) | 13 (6%) |
| Food court | Less than once a month | 432 (52%) | 79 (32%) | 76 (37%) |
|  | 1-2 times | 269 (32%) | 88 (35%) | 64 (31%) |
|  | 3-5 times | 96 (12%) | 59 (24%) | 42 (21%) |
|  | > 6 times | 40 (5%) | 25 (10%) | 22 (11%) |

Table S1: Response, by three categories of food security, frequencies and proportions for dining out questions

| **Outcome** | **Category** | **High-Marginal Food Security** | **Low Food Security** | **Very Low Food Security** |
| --- | --- | --- | --- | --- |
| **Please indicate the level of importance for the following features of a food court:** | |  |  |  |
| Convenient location | Important | 361 (90%) | 126 (73%) | 100 (78%) |
|  | Neither important nor unimportant | 40 (9%) | 35 (21%) | 20 (16%) |
|  | Unimportant | 4 (1%) | 11 (6%) | 8 (6%) |
| Cultural familiarity with food options | Important | 179 (44%) | 89 (52%) | 59 (46%) |
|  | Neither important nor unimportant | 172 (43%) | 64 (37%) | 48 (38%) |
|  | Unimportant | 54 (13%) | 19 (11%) | 21 (16%) |
| Affordability | Important | 358 (88%) | 135 (79%) | 112 (88%) |
|  | Neither important nor unimportant | 37 (9%) | 34 (20%) | 12 (9%) |
|  | Unimportant | 10 (3%) | 3 (1%) | 4 (3%) |
| Speed of service | Important | 345 (85%) | 127 (74%) | 105 (82%) |
|  | Neither important nor unimportant | 52 (13%) | 40 (23%) | 17 (13%) |
|  | Unimportant | 8 (2%) | 5 (3%) | 6 (5%) |
| Whatever is most convenient | Important | 201 (50%) | 89 (52%) | 75 (59%) |
|  | Neither important nor unimportant | 180 (44%) | 68 (39%) | 40 (31%) |
|  | Unimportant | 24 (6%) | 15 (9%) | 13 (10%) |
| Something inexpensive | Important | 265 (65%) | 117 (68%) | 90 (70%) |
|  | Neither important nor unimportant | 112 (28%) | 47 (27%) | 28 (22%) |
|  | Unimportant | 28 (7%) | 8 (5%) | 10 (8%) |
| Value for money | Important | 357 (88%) | 129 (75%) | 108 (84%) |
|  | Neither important nor unimportant | 42 (10%) | 36 (21%) | 13 (10%) |
|  | Unimportant | 6 (2%) | 7 (4%) | 7 (6%) |
| Recognised brand | Important | 190 (47%) | 93 (54%) | 71 (56%) |
|  | Neither important nor unimportant | 171 (42%) | 69 (40%) | 44 (34%) |
|  | Unimportant | 44 (11%) | 10 (6%) | 13 (10%) |
| **On average, how much do you spend per person on one trip to a food court?** | $0-10 | 149 (37%) | 45 (26%) | 37 (29%) |
|  | $11-20 | 217 (54%) | 86 (50%) | 60 (47%) |
|  | $21-30 | 31 (7%) | 26 (15%) | 19 (15%) |
|  | >$31 | 8 (2%) | 15 (9%) | 12 (9%) |

| **Outcome** | **Category** | **High-Marginal Food Security** | **Low Food Security** | **Very Low Food Security** |
| --- | --- | --- | --- | --- |
| **In an average week, how many dinner time meals do you cook::** |  |  |  |  |
| Using all raw produce e.g. fresh vegetables, un-prepared meats | Rarely | 96 (12%) | 25 (10%) | 25 (12%) |
|  | Less than once | 49 (6%) | 27 (11%) | 21 (10%) |
|  | 1-2 times | 123 (15%) | 59 (24%) | 49 (24%) |
|  | 3-4 times | 222 (27%) | 64 (26%) | 48 (24%) |
|  | 5-6 times | 213 (25%) | 45 (18%) | 40 (20%) |
|  | Everyday | 134 (16%) | 31 (12%) | 21 (10%) |
| Using a mixture of pre-packaged and fresh (raw) produce? e.g. fresh meat, spaghetti and a can/bottle sauce. | Rarely | 117 (14%) | 24 (10%) | 20 (10%) |
|  | Less than once | 144 (17%) | 34 (14%) | 31 (15%) |
|  | 1-2 times | 324 (39%) | 108 (43%) | 84 (41%) |
|  | 3-4 times | 149 (18%) | 55 (22%) | 50 (25%) |
|  | 5-6 times | 66 (8%) | 23 (9%) | 12 (6%) |
|  | Everyday | 37 (4%) | 7 (3%) | 7 (3%) |
| Using only frozen pre-packaged products e.g only defrosting and heating required | Rarely | 412 (49%) | 68 (27%) | 65 (32%) |
|  | Less than once | 217 (26%) | 65 (26%) | 44 (22%) |
|  | 1-2 times | 136 (16%) | 71 (28%) | 58 (28%) |
|  | 3-4 times | 46 (6%) | 31 (12%) | 24 (12%) |
|  | 5-6 times | 16 (2%) | 11 (4%) | 7 (3%) |
|  | Everyday | 10 (1%) | 5 (2%) | 6 (3%) |
| Using fresh, but pre-prepared produce e.g. meat with spices or marinade or meat-filled pasta from the refrigerated section | Rarely | 381 (46%) | 66 (26%) | 67 (33%) |
|  | Less than once | 213 (25%) | 65 (26%) | 55 (27%) |
|  | 1-2 times | 165 (20%) | 66 (26%) | 45 (22%) |
|  | 3-4 times | 48 (6%) | 38 (15%) | 25 (12%) |
|  | 5-6 times | 18 (2%) | 9 (4%) | 8 (4%) |
|  | Everyday | 12 (1%) | 7 (3%) | 4 (2%) |
| **How healthy is your diet?** | Unhealthy | 152 (18%) | 72 (29%) | 66 (33%) |
|  | Healthy | 685 (82%) | 178 (71%) | 137 (67%) |

| **Outcome** | **Category** | **High-Marginal Food Security** | **Low Food Security** | **Very Low Food Security** |
| --- | --- | --- | --- | --- |
| **I make more of an effort when cooking for:-** |  |  |  |  |
| Celebrations/Anniversary | Never | 91 (11%) | 31 (12%) | 21 (10%) |
|  | Rarely | 73 (9%) | 22 (9%) | 37 (18%) |
|  | Sometimes | 242 (29%) | 79 (32%) | 58 (28%) |
|  | Often | 211 (25%) | 70 (28%) | 44 (22%) |
|  | Always | 220 (26%) | 49 (20%) | 44 (22%) |
| Guests | Never | 70 (8%) | 20 (8%) | 15 (7%) |
|  | Rarely | 58 (7%) | 26 (10%) | 25 (12%) |
|  | Sometimes | 221 (26%) | 76 (30%) | 65 (32%) |
|  | Often | 238 (28%) | 80 (32%) | 53 (026%) |
|  | Always | 250 (30%) | 49 (20%) | 46 (23%) |
| **There are other occasions that I make more of an effort when cooking.** | Agree | 485 (58%) | 125 (50%) | 94 (46%) |
|  | Disagree | 352 (42%) | 126 (50%) | 110 (54%) |
| **On average, how much time do you spend on preparing your dinner meal? :** |  |  |  |  |
| Weekends | < 15 minutes | 97 (11%) | 21 (8%) | 19 (9%) |
|  | 15-30 minutes | 249 (30%) | 78 (31%) | 67 (33%) |
|  | 30-45 minutes | 249 (30%) | 94 (38%) | 72 (35%) |
|  | 45- 60 minutes | 168 (20%) | 33 (13%) | 33 (17%) |
|  | > 60 minutes | 74 (9%) | 25 (10%) | 13 (6%) |
| Week days | < 15 minutes | 98 (12%) | 20 (8%) | 21 (10%) |
|  | 15-30 minutes | 258 (31%) | 74 (30%) | 61 (30%) |
|  | 30-45 minutes | 280 (33%) | 105 (42%) | 75 (37%) |
|  | 45-60 minutes | 153 (18%) | 36 (14%) | 37 (18%) |
|  | > 60 minutes | 48 (6%) | 16 (6%) | 10 (5%) |
| **Rate your level of agreement** |  |  |  |  |
| I enjoy cooking | Strongly disagree | 52 (6%) | 11 (4%) | 13 (6%) |
|  | Disagree | 115 (14%) | 34 (14%) | 28 (14%) |
|  | Neither agree nor disagree | 234 (28%) | 84 (34%) | 49 (24%) |
|  | Agree | 322 (39%) | 87 (35%) | 80 (39%) |
|  | Strongly agree | 114 (14%) | 35 (14%) | 34 (17%) |

Table S2: Response, by three categories of food security, frequencies and proportions for dining in question

| **Outcome** | **Category** | **High-Marginal Food Security** | **Low Food Security** | **Very Low Food Security** |
| --- | --- | --- | --- | --- |
| **Rate your level of agreement** |  |  |  |  |
| I cook for sake of eating | Strongly disagree | 80 (10%) | 18 (7%) | 17 (8%) |
|  | Disagree | 195 (23%) | 60 (24%) | 30 (15%) |
|  | Neither agree nor disagree | 241 (29%) | 73 (29%) | 60 (29%) |
|  | Agree | 264 (32%) | 86 (34%) | 76 (37%) |
|  | Strongly agree | 57 (7%) | 14 (6%) | 21 (10%) |
| I feel cooking is a core | Strongly disagree | 72 (9%) | 13 (5%) | 13 (6%) |
|  | Disagree | 233 (28%) | 52 (21%) | 44 (22%) |
|  | Neither agree nor disagree | 264 (32%) | 102 (41%) | 73 (36%) |
|  | Agree | 212 (25%) | 67 (27%) | 55 (27%) |
|  | Strongly agree | 56 (7%) | 17 (7%) | 19 (9%) |
| Cooking is an opportunity to bond with my family | Disagree | 154 (18%) | 47 (19%) | 48 (23%) |
|  | Neither agree nor disagree | 325 (39%) | 92 (37%) | 67 (33%) |
|  | Agree | 358 (43%) | 112 (45%) | 89 (44%) |
| Cooking is an opportunity to bond with my friends | Strongly disagree | 51 (6%) | 10 (4%) | 11 (5%) |
|  | Disagree | 140 (17%) | 37 (15%) | 45 (22%) |
|  | Neither agree nor disagree | 318 (38%) | 95 (38%) | 60 (29%) |
|  | Agree | 279 (33%) | 96 (38%) | 76 (37%) |
|  | Strongly agree | 49 (6%) | 13 (5%) | 12 (6%) |
| Cooking makes me feel good | Strongly disagree | 43 (5%) | 10 (4%) | 12 (6%) |
|  | Disagree | 103 (12%) | 29 (12%) | 27 (13%) |
|  | Neither agree nor disagree | 277 (33%) | 91 (36%) | 57 (28%) |
|  | Agree | 316 (38%) | 89 (36%) | 82 (40%) |
|  | Strongly agree | 98 (12%) | 32 (13%) | 26 (13%) |
| Cooking is important to me because I know exactly what I am eating | Disagree | 84 (10%) | 32 (13%) | 25 (12%) |
|  | Neither agree nor disagree | 221 (26%) | 77 (31%) | 53 (26%) |
|  | Agree | 532 (64%) | 142 (57%) | 126 (62%) |
